# 3D Image Analysis of the Complete Ventricular-Subventricular Zone Stem Cell Niche Reveals Significant Vasculature Changes and Progenitor Deficits in Males Versus Females with Aging

**DOI:** 10.1016/j.stemcr.2021.03.012

**Published:** 2021-04-08

**Authors:** Xiuli Zhao, Yue Wang, Eric Wait, Walt Mankowski, Christopher S. Bjornsson, Andrew R. Cohen, Kristen L. Zuloaga, Sally Temple

**Affiliations:** 1 Neural Stem Cell Institute, Rensselaer, NY 12144, USA; 2 Department of Neuroscience & Experimental Therapeutics, Albany Medical College, Albany, NY 12208, USA; 3 Department of Electrical and Computer Engineering, Drexel University, Philadelphia, PA 19104, USA; 4 Advanced Imaging Center, Howard Hughes Medical Institute Janelia Research Campus, Ashburn, VA 20147, USA; 5 Department of Radiology, Perelman School of Medicine, University of Pennsylvania, Philadelphia, PA 19103, USA

**Keywords:** sex, neural stem cells, niche, vascular, aging, neurogenesis, blood vessels, brain, subventricular zone

## Abstract

With age, neural stem cell (NSC) function in the adult ventricular-subventricular zone (V-SVZ) declines, reducing memory and cognitive function in males; however, the impact on females is not well understood. To obtain a global view of how age and sex impact the mouse V-SVZ, we constructed 3D montages after multiplex immunostaining, and used computer-based 3D image analysis to quantify data across the entire niche at 2, 18, and 22 months. We discovered dramatic sex differences in the aging of the V-SVZ niche vasculature, which regulates NSC activity: females showed increased diameter but decreased vessel density with age, while males showed decreased diameter and increased tortuosity and vessel density. Accompanying these vascular changes, males showed significant decline in NSC numbers, progenitor cell proliferation, and more disorganized migrating neuroblast chains with age; however, females did not. By examining the entire 3D niche, we found significant sex differences, with females being relatively spared through very old age.

## Highlights

• Aging impacts the mouse V-SVZ significantly more in males than females
• Vessel tortuosity, narrowness, and density increase in aged males not females
• Progenitor cell division and neuroblast chain organization decline in aging males
• Numbers of type B stem cells decline significantly in aged males not females

## Graphical abstract

**Figure:**
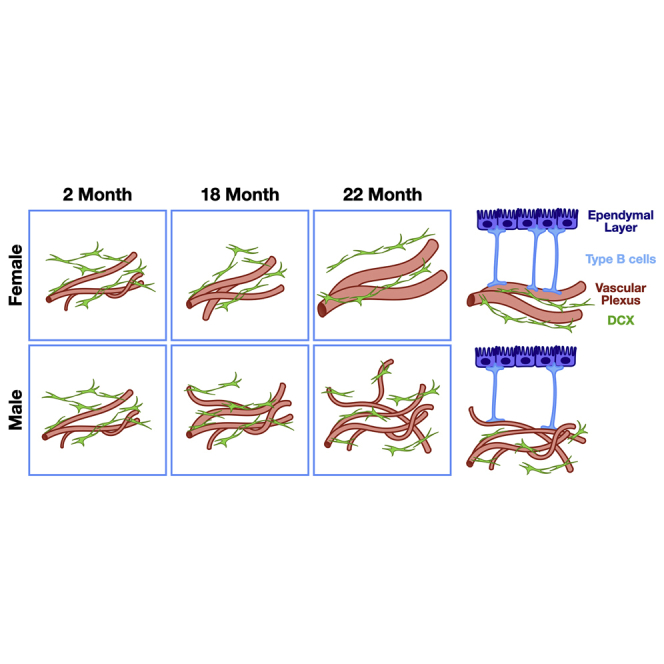

## Teaser

To explore sex differences with age in the adult mouse neural stem cell niche, Temple and colleagues analyzed entire 3D V-SVZ whole-mounts via computer-based multiplex image analysis. Sexual dimorphism was seen with aging in the vascular plexus, a critical niche component, along with more severe decline in neural stem cell number, progenitor proliferation and neuroblast organization in males compared with females.

## Introduction

The ventricular-subventricular zone (V-SVZ) harbors neural stem cells (NSCs) that replenish neurons throughout life in the olfactory bulb in the adult mouse brain. With age, there is a dramatic decline in V-SVZ neurogenesis accompanied by changes in the niche, including decreased thickness (Luo et al., 2006), loss of ependymal cell coverage (Conover and Shook, 2011), increased reactive phenotypes of ependymal cells and astrocytes (Capilla-Gonzalez et al., 2014), changes in the choroid plexus secretome (Silva-Vargas et al., 2016), and increased microglial activation (Solano Fonseca et al., 2016). Less well studied is the response of the specialized V-SVZ vasculature which, as in other stem cell niches, plays a vital role. Furthermore, these observations have been made in aging males, so response of the niche and NSC lineage to aging in females is not known.

Blood vessels within the V-SVZ are directly contacted by cells in the stem cell lineage, collectively called neural progenitor cells (NPCs). The type B NSCs are situated mostly at the ependymal surface and project a long process to contact the blood vessel surface within the V-SVZ vascular plexus (Mirzadeh et al., 2008). Type B cells produce transit amplifying type C cells that are closely apposed to the vasculature (Tavazoie et al., 2008). Type C cells in turn give rise to type A neuroblasts that move slightly away from the vascular surface but use this to guide their migration out of the V-SVZ and toward the olfactory bulbs (Bovetti et al., 2007). We and others have demonstrated that blood vessels in the V-SVZ niche preserve the pool of quiescent stem cells, and promote proliferation and neurogenesis (Kokovay et al., 2010; Ottone et al., 2014; Shen et al., 2008). In young male mice, vessels in the V-SVZ have increased density, lower tortuosity, and reduced blood flow compared with the surrounding tissue (Culver et al., 2013). However, how blood vessel parameters, such as diameter and tortuosity, change with age have not been examined in the V-SVZ.

Most previous studies have been performed in male animals, and the structure- and age-related changes in the V-SVZ niche in females have not been characterized. Age-related changes in sex hormones alter the function of the cerebrovasculature (Robison et al., 2019) and, notably, sex and sex hormones influence neurogenesis (Ponti et al., 2018). Since both sex hormones and neurogenesis decline with age (Apple et al., 2017; Barron and Pike, 2012), uncovering interactions between sex and aging in the vascularized NSC niche may be critical for understanding the regulation of adult neurogenesis and how declines may differentially affect the sexes.

The goal of this study was to determine if there are sex differences in V-SVZ aging, focusing on the vascular niche due to its physiological importance. To obtain a comprehensive view of the aging V-SVZ niche, we imaged, reconstructed, and analyzed entire 3D whole-mounts, and investigated sex differences in vessel structure, neuroblast chains, and NPC proliferation at age 2, 18, and 22 months. We discovered clear sex-dependent changes in the V-SVZ niche vasculature, as well as differences in the population dynamics of NPCs with age, with males showing significantly greater impairment in this stem cell system than females.

## Results

**Figure 1:**
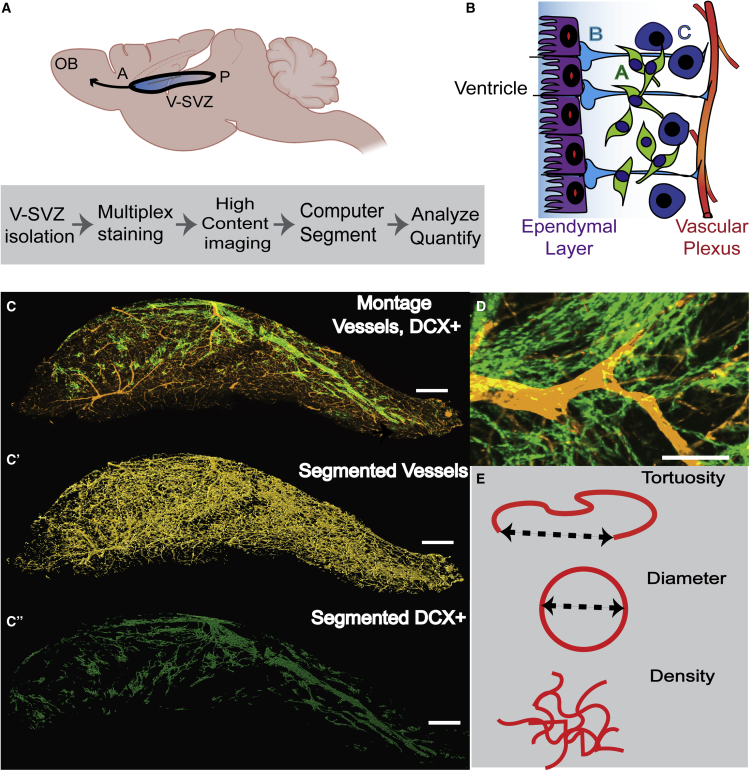
V-SVZ Whole-Mount Preparation, Labeling, Segmentation, and Analysis Parameters (A) Left: schematic of the location of the V-SVZ in the brain and image analysis process. (B) Diagram of the V-SVZ showing type B stem cells, type C transit amplifying cells, and type A neuroblasts, located between the ependymal layer and the vascular plexus. (C) Representative image (10x) of a V-SVZ whole mount labeled with anti-laminin (gold) to identify blood vessels, and anti-doublecortin (DCX) (green) to label neuroblasts. Scale bar, 300 μm. C′ and C″ segmentation results for the laminin+ (vessels) and the DCX+ channels, respectively. (D) High power image showing DCX+ chains in relation to blood vessels. Scale bar, 100 μm. (E) Schematics of each analysis parameter, and how calculated: for tortuosity and diameter, the dotted line represents the measured length/area. Density is defined as DCX volume/V-SVZ volume.

Understanding how a multifarious tissue changes with important biological variables, such as age and sex, is difficult with approaches that rely on sectioning, which provide a limited view of the tissue. Hence, we used a 3D whole-mount preparation, which allows us to study the entire V-SVZ niche as an explant that retains the key 3D inter-relationships of cells seen *in vivo* (Figure 1A).

### Whole-Mount Preparation, Labeling, and Segmentation

To determine if there are sex-dependent changes in the vascular V-SVZ niche with age, we dissected V-SVZ whole-mounts from male and female C57BL/6 mice at age 2, 18, and 22 months (Figure 1A) and examined the niche vascular plexus and associated progenitor cells in 3D renderings. Whole-mounts were stained with anti-laminin to label the basement membrane at the surface of the V-SVZ blood vessels, glial fibrillary acidic protein (GFAP) for type B NSCs, doublecortin (DCX) for type A neuroblasts, Ki67 for proliferating cells, beta catenin for ependymal cells, and 4′,6-diamidino-2-phenylindole (DAPI) for cell nuclei. z Stack images were acquired through the entire depth of the V-SVZ germinal zone.

After multiplex immunostaining, high-content images (approximately 50 per whole-mount) were generated and stitched together to create a complete montage of the 3D niche. Computer-based analysis of these large 3D images was performed by segmenting, thresholding, and quantifying features across the entire niche or within a designated region, such as the anterior aspect, where NSC activity is high in young animals. For analysis of vessels and neuroblast chains, 3D computer-assisted analysis was utilized to segment the vessels or chains to generate skeletons. Segmentation and reconstruction enabled a full view of the V-SVZ plexus in 3D with thousands of feature measurements per imaging channel per V-SVZ, and a minimum of four samples per age per sex. Representative images of immunolabeling, segmentation, and analysis parameters are shown in Figures 1, S1, and S2; Videos S1 and S2.

Video S1. Representative images of blood vessels labeled with laminin in whole-mount V-SVZs from 2-, 18-, and 22-month-old female and male mice

Video S2. Representative images of segmented GFAP in red, DCX in green and blood vessels in brown in whole-mount V-SVZs from 2-month-old female mice

### Tortuosity and Density of V-SVZ Blood Vessels Increases with Age in Males

**Figure 2:**
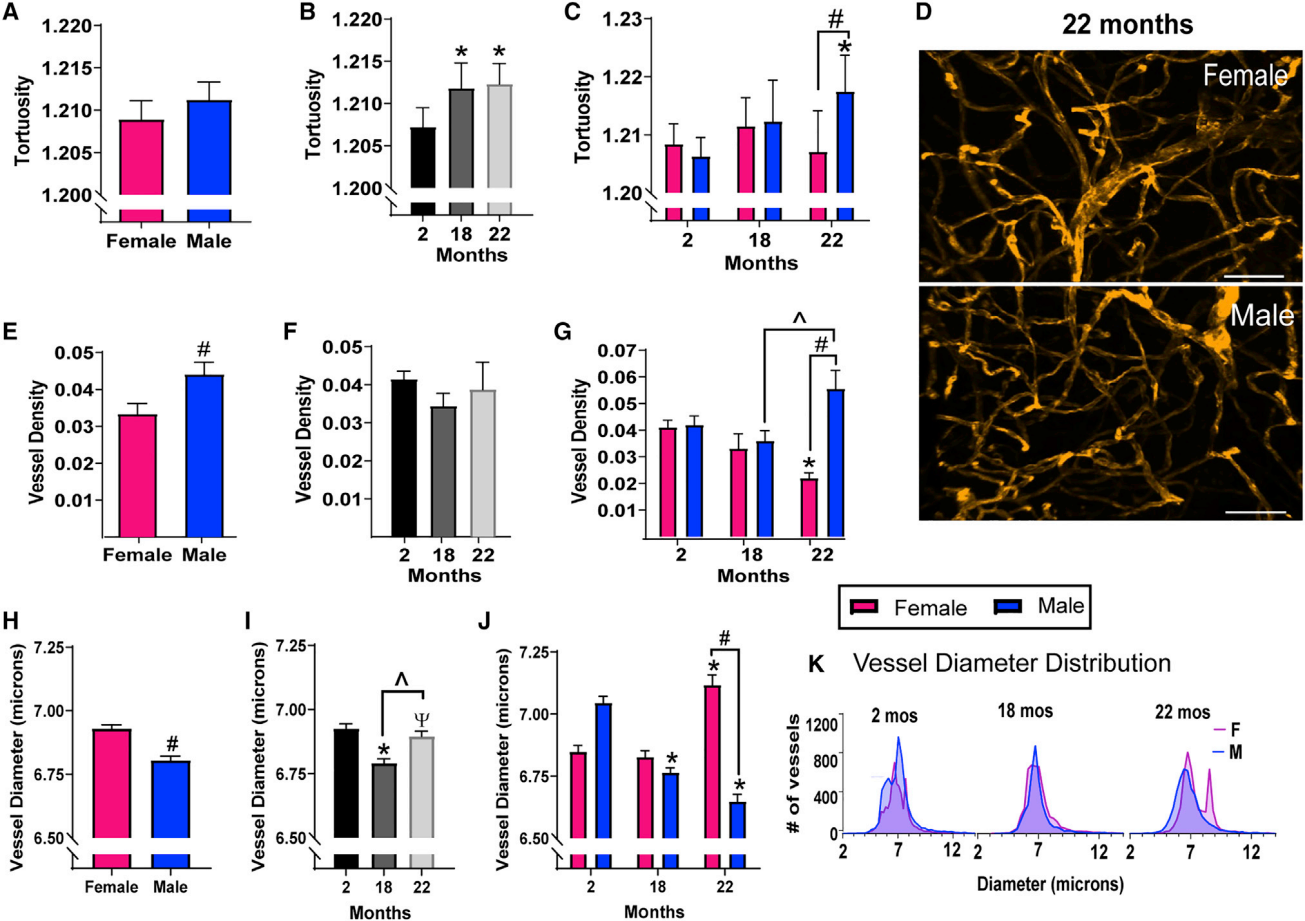
Tortuosity and Density of V-SVZ Blood Vessels Increases with Age in Males, while Vessel Diameter Decreases in Males and Increases in Females Vessel tortuosity, density, and diameter were calculated for the entire V-SVZ for each sample and displayed (A, E, and H) with ages combined; (B, F, and I) with sexes combined; and (C, G, and J) separated by age and sex. (D) Representative images of V-SVZ vessels in 22-month-old females and males. Scale bar, 50 μm. (A–G) ^∗^p < 0.01 versus 2-month-old mice of the same sex; #p < 0.0001 versus females of same age; ˆp < 0.05 versus 18 months. (H–J) ^∗^p < 0.0001 versus 2-month-old mice of the same sex; #p < 0.0001 versus females of the same age ; ˆp < 0.0001 versus 18 months; Ψp < 0.01 versus 2 months. Graphs show means ± SEM, N = 4–6 mice per group, statistical analysis by unpaired two-tailed Student's t test for (A), (E), and (H), one-way ANOVA with Tukey's multiple comparisons test for (B), (F), and (I), two-way ANOVA with Sidak's multiple comparisons test for (C), (G), and (J). (K) The distribution of blood vessel diameters across ages for males and females (p < 0.0001 for each age, Kolmogorov-Smirnov test). N = 3,685–6,638 vessels per group, N = 4–6 mice per group (2-month-old females, N = 6; 2-month-old males, N = 6; 18-month-old females, N = 5; 18-month-old males, N = 4; 22-month-old females, N = 4; 22-month-old males N = 4).

Previous studies have shown that blood vessels in the V-SVZ of young mice are more linear than those in the surrounding tissue (Culver et al., 2013); however, cerebral vessel tortuosity increases with age in other brain regions, including in humans (Thore et al., 2007). Therefore, we hypothesized that increased vessel tortuosity may occur with age in the V-SVZ. To measure how tortuous the vessels were, we calculated the ratio between the measured path length of the blood vessel, which includes twists and turns, and the most direct path length (a straight line) (Figure 1E). Laminin-labeled blood vessels in the whole-mounts of the V-SVZ of 2-, 18-, and 22-month-old males and females (n = 4–6 per group) showed no sex differences in tortuosity when age was not taken into account (Figure 2A). When age was considered and sexes were combined, tortuosity was increased at 18 and 22 months compared with 2 months (p < 0.01; Figure 2B). Notably, when mice were separated by sex and age, interactions between these factors were identified. In males, there was an increase in tortuosity with age that reached significance at 22 months (p < 0.0001 versus 2-month-old males), while no significant change with age was seen in females. At 22 months, males have significantly more tortuous vessels than females (p < 0.0001 versus 22-month-old females; Figures 2C and 2D).

Given observations that young male mice have higher blood vessel density in the V-SVZ than in the surrounding tissue (Culver et al., 2013), we examined the density of blood vessels defined as the ratio of volume occupied by vessels to the total volume of the V-SVZ. When mice were pooled by sex, regardless of age, males showed higher vessel density than females (p < 0.05; Figure 2E). When both sexes were pooled together, important age-related changes were again masked (Figure 2F). Separating by sex, we found that V-SVZ vessel density increased at 22 months of age in males (p < 0.05 versus 18-month-old males) but decreased at 22 months of age in females (p < 0.05 versus 2-month-old females), resulting in greater vessel density in males compared females at 22 months (p < 0.0001; Figures 2D and 2G).

### V-SVZ Vessel Diameter Varies with Age in a Sex-Dependent Manner

Cell proliferation in the V-SVZ is associated with a local increase in cerebral blood flow (Lacar et al., 2012). Since blood flow is inversely proportional to vessel diameter, we sought to determine if V-SVZ vessel diameter might also change with age. Diameter was assessed from segmentations of laminin-stained vessels, as described above (Figure 1E). Again, pooling by sex or age alone masked important differences (Figures 2H and 2I), but separating mice by sex and age revealed sex-dependent changes with aging (Figure 2J). We found that V-SVZ vessel diameter steadily decreased with age in males (p < 0.0001 for 18- and 22-month-old males versus 2-month-old males). Conversely, diameter sharply increased in females at age 22 months (p < 0.0001 versus 2- or 18-month-old females). These changes resulted in females having significantly larger vessel diameters than males at 22 months (p < 0.0001; Figure 2J).

Vessel diameter distribution also differed between males and females at 2, 18, and 22 months (p < 0.0001 for each age, Kolmogorov-Smirnov test; Figures 2J–2K). In both sexes, at all ages, the vast majority of vessels in the V-SVZ were <10 μm in diameter, defining them as capillaries. The average capillary size was approximately 7 μm, and in 22-month-old females these vessels increase in number and a new population of larger capillaries of 8.25 μm mean diameter appears that is not obvious in other groups (Figure 2K).

### NSC and NPC Population and Proliferation Changes *in vivo* with Age and Sex

**Figure 3:**
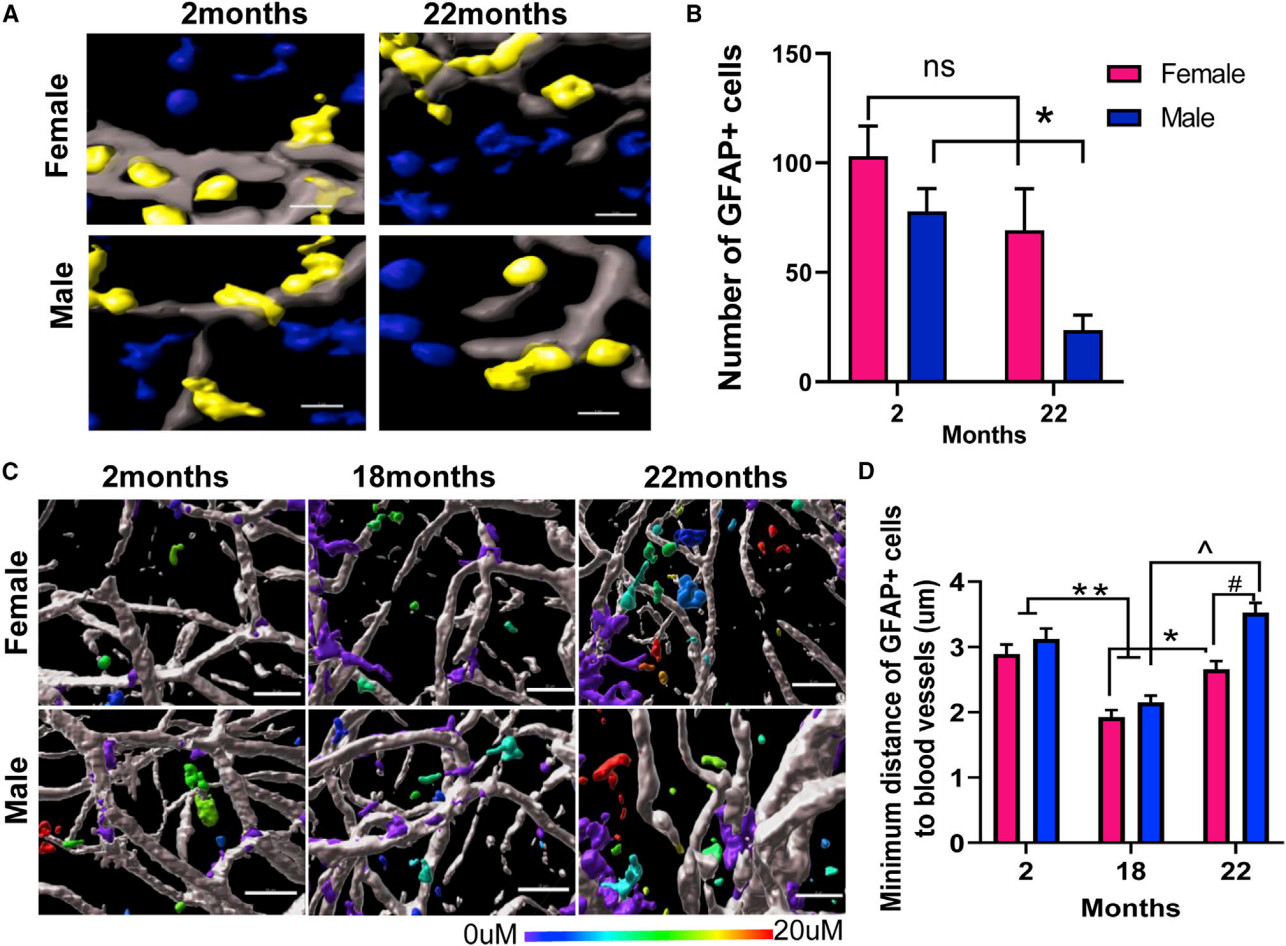
Quantifying Apical GFAP+ Type B and their Distance to the Nearest Blood Vessel Surface Reveals Significant NSC Loss in Males but not Females with Aging (A) Representative images of segmented GFAP+ cells in the V-SVZ whole-mount. The GFAP+ cells are defined by colocalizing DAPI+-stained nuclei within GFAP+ cells using automated image analysis for unbiased quantification. GFAP staining is shown in gray, DAPI nuclei that are colocalized with GFAP are shown in yellow, while those not colocalized are shown in blue. Scale bar, 5 μm. (B) The number of apical GFAP+ cells in males and females at 2 and 22 months. ^∗^p < 0.05 2- versus 22-month-old males. (C) Representative images showing the minimum distance between GFAP+ cells and laminin+ blood vessels with age and sex. Scale bar, 25 μm. Distances range from 0 to 20 μm and distances change with age as quantified. (D) ^∗∗^p < 0.0001 2- versus 18-month-old mice of both sexes; ^∗^p < 0.01 18- versus 22-month-old females; ˆp < 0.0001 18- versus 22-month-old males; #p < 0.0001 males versus females of the same age). N = 1,111–1,630 segmented GFAP+ cells per group (2-month-old females, N = 1,111; 2-month-old males, N = 1,167; 18-month-old females, N = 1,177; 18-month-old males, N = 1,630; 22-month-old females, N = 1,517; 22-month-old males, N = 1,468). Segmented GFAP+ cells were quantified from 3 mice per group with 3 images per mouse. Two-way ANOVA with Tukey's multiple comparisons test.

Our previous studies demonstrated that apical GFAP+ type B NSCs form loose sheets of cells directly beneath the ependymal surface while frequently having an apical process penetrating through the ependymal layer and a basal process contacting the V-SVZ blood vessels (Figure 1B). Given the importance of the vascular niche to NSC maintenance and neurogenesis, we quantified these apical GFAP+ type B cells (situated within 5 μm of the ependymal layer) to determine if the NSC population is affected by age and sex. Previous studies demonstrated a decline in V-SVZ NSC number at 20–26 months in males (Ahlenius et al., 2009; Jin et al., 2003). We used computer-based analysis that assessed thousands of cells in an unbiased manner to identify GFAP+ cells within 5 μm of the ependymal surface. For each image, we quantified the number of type B cells in males and females, using the overlap of DAPI-stained nuclei with GFAP staining to identify type B cells. Corroborating these previous findings, we found that apical type B cells decreased significantly in males from 2 to 22 months (Figures 3A and 3B, p < 0.05). In contrast, unexpectedly, the number of apical type B cells in females did not significantly change with age (Figures 3A and 3B, 2- versus 22-month-old mice, p = 0.2959). Hence the age-related decline in NSCs well documented in previous studies appears to be sex specific, occurring in males and not obvious in females.

Previously, we reported that neurogenesis is associated with SVZ blood vessels and that activated, proliferating NSCs lie close to the niche vasculature (Shen et al., 2008). Hence, we analyzed the distribution of GFAP+ cells relative to the nearest blood vessel surface, focusing on three anterior regions, using automated image analysis (Figure S2). Representative images showing the minimum distance between GFAP+ cells and the nearest blood vessel surface are indicated with color coding (Figure 3C). In both sexes, the distance to the nearest blood vessel surface decreased from 2 to 18 months (^∗∗^p < 0.0001 versus 2-month-old mice of the same sex), but increased at 22 months (^∗^p < 0.01 18- versus 22-month-old females; ˆp < 0.0001 18- versus 22-month-old males) (Figure 3D). GFAP+ cells were significantly further away from the vessels in the 22-month-old males versus females, V-SVZ (#p < 0.0001). Hence, there are complex age- and sex-dependent differences in proximity of GFAP+ cells to niche blood vessels.

**Figure 4:**
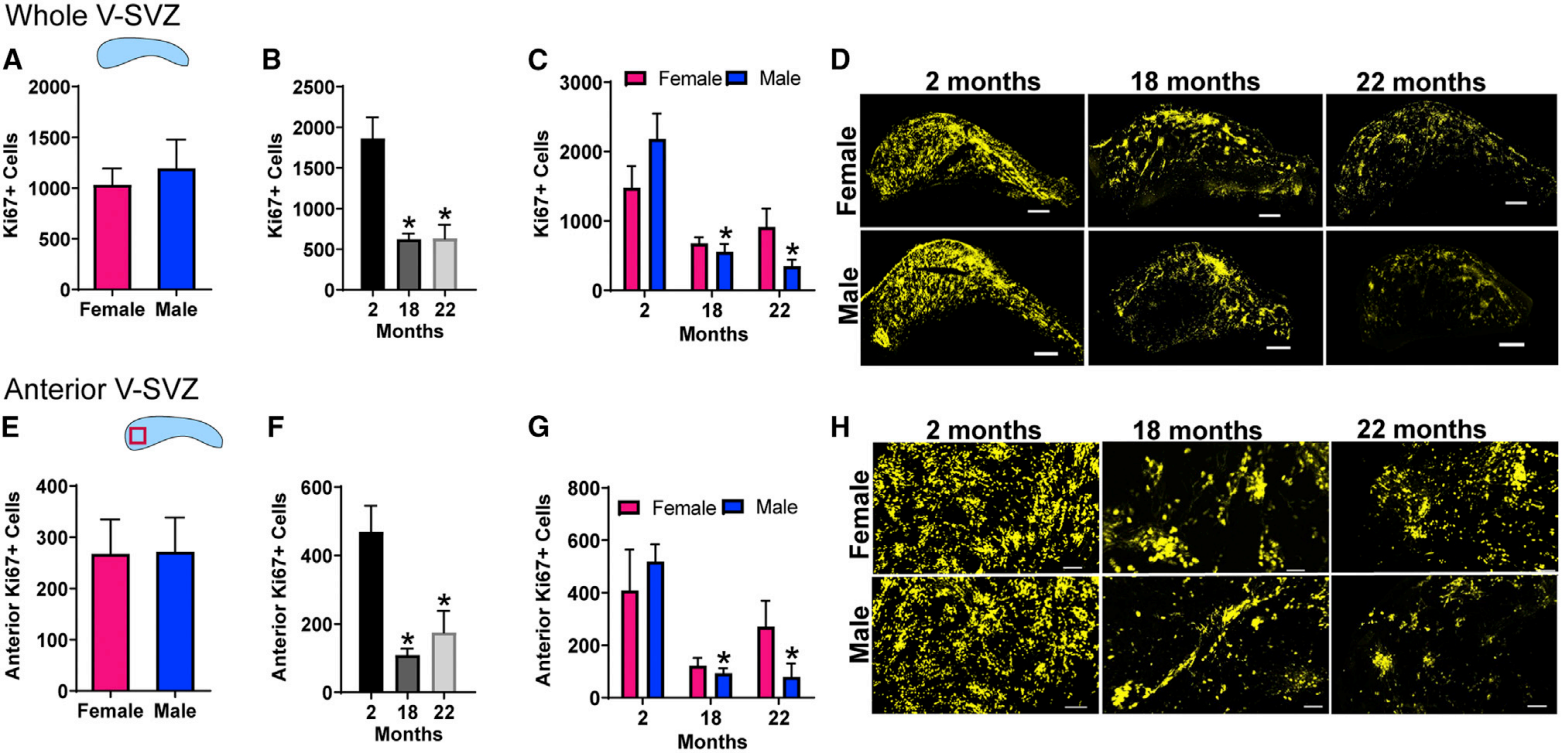
NPC Proliferation *in vivo* is Affected by Age and Sex Immunostaining for the proliferation marker Ki67 was quantified in the whole V-SVZ (A–D) and just the anterior portion (E–H) and displayed as (A and E) with ages combined, (B and F) with sexes combined, and (C and G) separated by age and sex. Representative images of NPC proliferation in the whole (D) and the anterior area of the V-SVZ (H). N = 4–6 per group (2-month-old females, N = 5; 2-month-old males, N = 6; 18-month-old females, N = 5; 18-month-old males, N = 4; 22-month-old females, N = 4; 22-month-old males, N = 4), ^∗^p < 0.01 versus 2-month-old mice of the same sex. Graphs show mean ± SEM, statistical analysis by unpaired two-tailed Student's t test for (A and E), one-way ANOVA with Tukey's multiple comparisons test for (B and F), and two-way ANOVA with Sidak's multiple comparisons test for (C and G).

To assess overall NPC proliferation, we examined the number of Ki67+ cells in the V-SVZ whole mounts. The number did not vary by sex when ages were combined (Figure 4A) but declined at both 18 and 22 months compared with 2 months when sexes were combined (p < 0.001 at each age; Figure 4B). However, this age-related decrease in Ki67+ cells reached statistical significance in the males (p < 0.001 2-month-old males versus 18- or 22-month-old males) but not in the females (Figure 4C) (representative images are shown in Figure 4D). Similar results were obtained when we focused on the anterior V-SVZ, a region of active NPC activity in young animals (Coskun et al., 2007) (p < 0.01 2-month-old males versus 18- or 22-month-old males, Figures 4E–4G, and representative images of the region are shown in Figure 4H).

### Density and Organization of DCX Neuroblast Chains with Age

**Figure 5:**
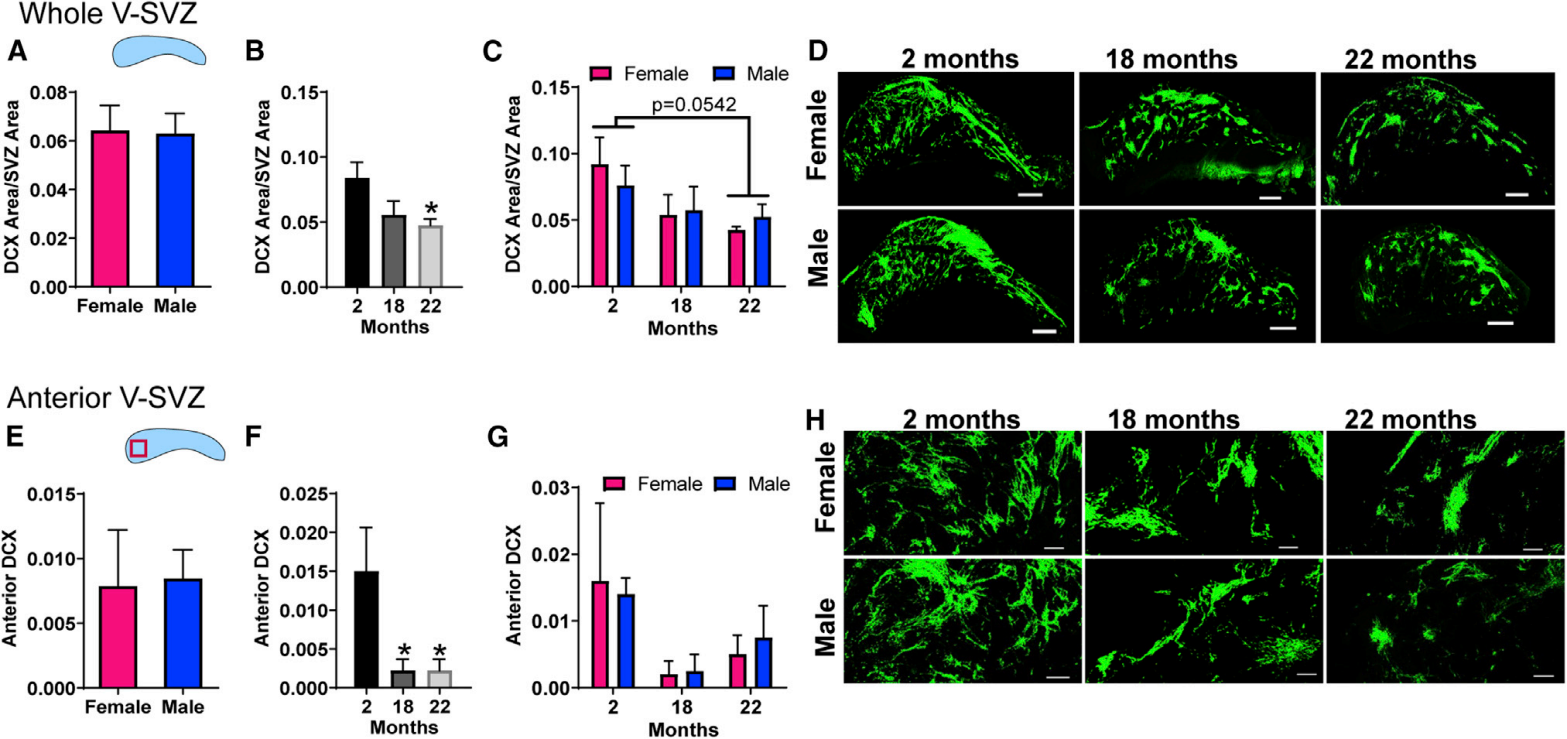
Density of Neuroblast Chains Decreases with Age in both Sexes Neuroblast chain density was calculated as DCX voxels/total V-SVZ voxels (A–D) and for the anterior V-SVZ (E–H) for each sample and displayed (A and E) with ages combined, (B and F) with sexes combined, and (C and G) separated by age and sex. Representative images of DCX neuroblast chain in the whole (D) and the anterior area of the V-SVZ (H). N = 4–6 per group (2-month-old females, N = 5; 2-month-old males, N = 5; 18-month-old females, N = 5; 18-month-old males, N = 4; 22-month-old females, N = 4; 22-month-old males, N = 4), ^∗^p < 0.05 versus 2-month-old mice. Graphs show mean ± SEM, statistical analysis by unpaired two-tailed Student's t test for (A and E), one-way ANOVA with Tukey's multiple comparisons test for (B and F), and two-way ANOVA with Sidak's multiple comparisons test for (C and G).

To obtain a global assessment of neurogenesis, we assessed production of DCX+ type A cells. These form chains of migrating cells prominently in the dorsal aspect of the V-SVZ. DCX chain density did not vary by sex when ages were combined (Figure 5A), but when sexes were combined, it decreased at 22 months (p < 0.05 versus 2-months; Figure 5B). No sex-related differences were found with age (Figure 5C) (representative images are shown in Figure 5D). The decrease in DCX chain density was most pronounced in the anterior portion of the V-SVZ (Figures 5E–5G), at both 18 and 22 months (p < 0.05 versus 2 months; Figure 5F) (representative images of the anterior region are shown in Figure 5H).

**Figure 6:**
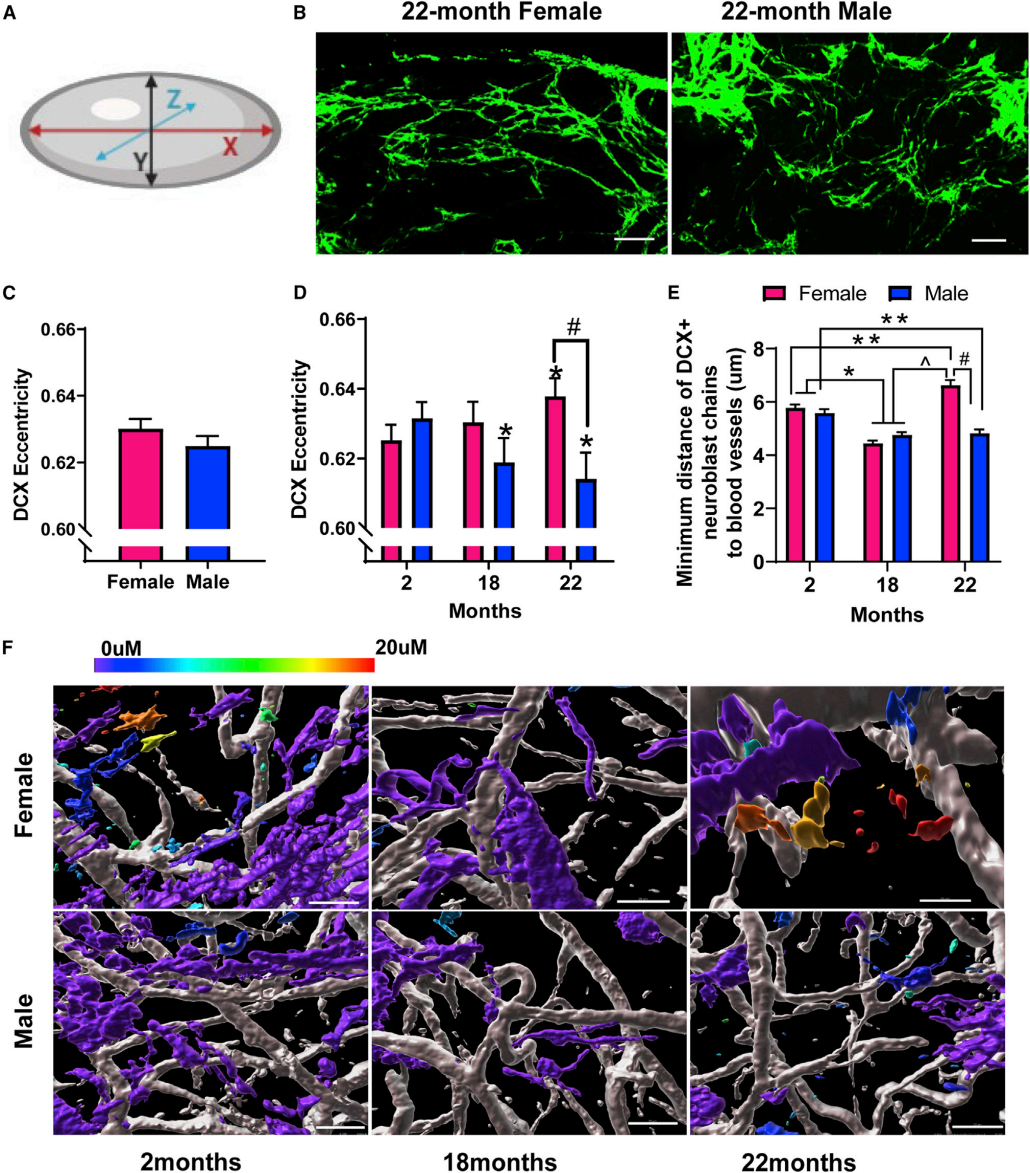
Organization of Neuroblast Chains Decreases with Age in Males (A) Schematic: eccentricity was measured in 3D by calculating 1 (the ratio between the middle principal component and the maximal principal component). An eccentricity of 1 indicates a line and an eccentricity of 0 indicates a circle. (B) Representative images of DCX staining in the dorsal side of the V-SVZ whole mount from 22-month-old females and males. Scale bar, 100 μm. (C) Eccentricity of the DCX chains with ages combined. (D) Eccentricity separated by age and sex. N = 4–6 per group (2-month-old females, N = 5; 2-month-old males, N = 5; 18-month-old females, N = 5; 18-month-old males, N = 4; 22-month-old females, N = 4; 22-month-old males, N = 4), ^∗^p < 0.05 versus 2-month-old mice of the same sex; #p < 0.0001 versus females of the same age. (E) DCX+ neuroblast chains and blood vessels were segmented, three images per mouse, three mice per group; distance from chain surface to blood vessel surface was calculated, separated by age and sex (^∗^p < 0.0001 2- versus 18-month-old mice of the same sex; ˆp < 0.0001 18- versus 22-month-old females; ^∗∗^p < 0.01 2- versus 22-month-old mice of the same sex; #p < 0.0001 females versus males at 22 months). N = 1,119–2,202 segmented DCX+ neuroblast chains per group (2-month-old females, N = 2,056; 2-month-old males, N = 1,823; 18-month-old females, N = 2,180; 18-month-old males, N = 2,202; 22-month-old females, N = 1,119; 22-month-old males, N = 1,456). Two-way ANOVA with Tukey's multiple comparisons test. (F) Representative images showing the minimum distance between DCX+ chains and laminin+ blood vessels color coded to show distance. Scale bar, 25 μm.

Given the changes in vessel tortuosity we observed in aged males, and the tendency of neuroblasts to migrate along blood vessels, we hypothesized that aged males might also have more disorganized DCX chains. In 3D, chain linearity can be measured by eccentricity, calculated as one minus the ratio between the middle and maximal principal components of an object: the lower the value, the less eccentric the chains, meaning they are less linear and more disorganized (Figure 6A) (representative images are shown in Figure 6B). When mice were not separated by age, important sex-dependent effects were masked (Figure 6C). However, when mice were separated by age and sex, the eccentricity of DCX chains decreased in 18-month-old males (p < 0.01) and 22-month-old males (p < 0.0001) compared with 2-month-old males (Figure 6D). Conversely, in females, eccentricity increased with age ((p < 0.001 2 versus 22 months; p < 0.05 18 versus 22 months); Figure 6D). By age 22 months, males had significantly decreased eccentricity indicating greater disorganization of the chains compared with females (p < 0.0001; Figure 6D), supporting our hypothesis that neuroblast migration would be more disrupted in aged males given their greater vessel tortuosity.

To determine if there are age- and/or sex-related differences in the interaction between NPCs and niche blood vessels, we assessed the distance between DCX+ cells and the closest vessel surface using the method shown in Figure S2. Given that DCX+ neuroblasts migrate in chains, we first segmented the chains and the vessels, then calculated the minimum distance to blood vessels from the chain (object) surface. While there was no difference between females and males at 2 and 18 months, sex differences emerged at 22 months (#p < 0.0001 females versus males at 22 months; Figure 6E). Interestingly, DCX+ cells were closest to the nearest vessel surface at 18 months in females, then moved further away at 22 months (^∗^p < 0.0001 2- versus 18-month-old females; ˆp < 0.0001 18- versus 22-month-old females; Figure 6E). In contrast, males showed reduced distance between DCX+ cells and the blood vessel surface with age (^∗^p < 0.0001 2 versus 18 months, ^∗∗^p < 0.01 2 versus 22 months; Figure 6E). Representative images of the minimum distance indicated by color coding are shown in Figure 6F.

### NPC Proliferation Changes *in vitro* with Age and Sex

We previously demonstrated that transit amplifying type C cell proliferation is dysregulated with age, leading, surprisingly, to more MASH1+ type C cells in the V-SVZ of 22-month-old compared with 18-month-old mice *in vivo*. This behavior was also observed when the NPCs were removed from the niche and cultured, indicating a cell-autonomous change: there was more type C progenitor proliferation *in vitro* at 22 months than at 18 months (Apostolopoulou et al., 2017). However, the type C cell proliferation appeared to be non-productive as it did not result in more DCX+ neuroblasts. This indicated that type C proliferation was abnormally activated at 22 months. These observations were made in male animals, and age-related changes in NPC proliferation in females have not been examined.

**Figure 7:**
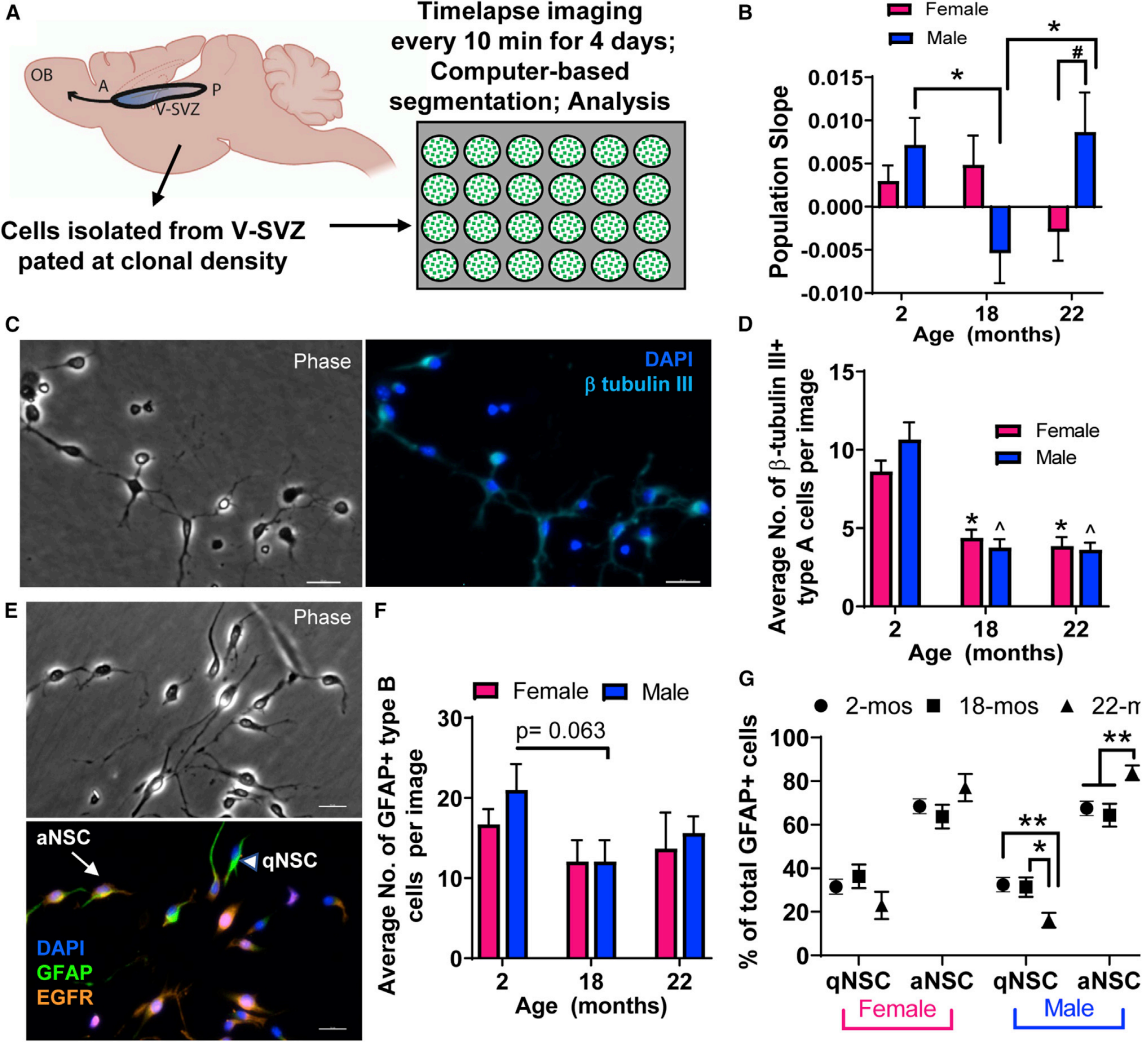
NPC Proliferation Differences *in vitro* with Age and Sex (A) Schematic: V-SVZ NPCs were isolated from male and female mice at age 2, 18, and 22 months, plated at clonal density, and time-lapse recorded for 4 days, then computer-based analysis performed. (B) Population growth separated by age and sex. N = 315 movies analyzed (2-month-old females, 70 movies; 2-month-old males, 56 movies; 18-month-old females, 48 movies; 18-month-old males, 45 movies; 22-month-old females, 48 movies; 22-month-old males, 48 movies; 3–4 experiments per group with 3 mice pooled per experiment, i.e., 9–12 mice per group). ^∗^p < 0.05 versus 2-month-old mice of the same sex; #p < 0.05 sex difference. Two-way ANOVA with Tukey's multiple comparisons test. (C–G) At the end of the time-lapse recording, cells were fixed, immunostained, and quantified: (C) representative images showing β-tubulin III+ staining of type A cells, image from 2-month-old male mice. Scale bar, 50 μm. (D) Quantification of β-tubulin III+ type A cells generated (^∗^p < 0.001; ˆp < 0.0001 versus 2-month-old mice of the same sex); (E) representative images showing cells stained with anti-GFAP (green) and anti-EGFR (orange) antibodies from 2-month-old male mice; DAPI-labeled cell nuclei shown in blue. Scale bar, 50 μm. Arrow points to a GFAP+ EGFR+ aNSC, arrowhead points to a GFAP+ EGFR− qNSC; (F) average number of GFAP+ type B cells per image; (G) percentage of GFAP+ EGFR+ aNSCs and GFAP+ EGFR− qNSCs generated at each age for males and females; ^∗^p < 0.05, ^∗∗^p < 0.01; two-way ANOVA with Tukey's multiple comparisons test. N = 206 images analyzed with 21–42 images per group (2-month-old females, 41 images; 2-month-old males, 42 images; 18-month-old females, 31 images; 18-month-old males, 32 images; 22-month-old females, 21 images; 22-month-old males, 39 images; 3 experiments per group with 3 mice pooled per experiment, total of 9 mice per group).

To address this, we isolated V-SVZ NPCs from male and female mice at 2, 18, and 22 months of age and grew them at low density and followed progenitor behavior with continuous time-lapse recording (Figure 7A). We produced 315 time-lapse movies, each of 980 image frames captured every 5 min over a period of 4 days. After segmenting and tracking cells in each image frame, a linear regression that fitted the number of cells per image frame was used to generate a slope value representing population growth. This robust “population slope” feature captures an overview of cell divisions and death by unbiased analysis. Similar to our previous findings, population growth was decreased in males at age 18 months but increased at 22 months (p < 0.05 for both versus 2-month-old males). However, in females, population growth did not significantly differ across age groups, i.e., the non-monotonic change observed in males was not detected. As a result, males showed greater population growth indicating greater division rate than females at 22 months (p < 0.05; Figure 7B).

Next, to determine the cell subtypes responsible for the population changes, after time-lapse recording the cells were immunostained and quantified for β-tubulin III for type A neuroblasts, GFAP for type B NSCs, further subdivided into activated (aNSCs) (GFAP+ EGFR+) and quiescent (qNSCs) (GFAP+ EGFR−) (Codega et al., 2014). There was an age-related decrease in production of type A neuroblasts in both sexes (Figures 7C and 7D, ^∗^p < 0.001, ˆp < 0.0001 versus 2-month-old mice of the same sex). Representative images of cells immunolabeled for GFAP and EGFR are shown in Figure 7E. The number of GFAP+ cells produced showed a decreasing trend from 2 to 18 months in males (p = 0.063; Figure 7F). The proportions of aNSCs and qNSCs in females did not significantly change with age. In males, however, there was a decrease in qNSCs (p < 0.01 2 versus 22 months; p < 0.05 18 versus 22 months; Figure 7G) and a significant increase in aNSCs produced in the cultures with age (p < 0.01 versus 2 and 18 months; Figure 7G).

Hence, upon isolation from the niche, 22-month-old males show increased NSC activation and increased NPC proliferation, but without increased neuron production, indicating abnormal proliferation. These changes are not seen in females, suggesting there are cell-autonomous differences in proliferative state in male versus female NSC lineages with advanced age, providing additional evidence for more profound deficits in males.

## Discussion

The adult V-SVZ has important features that benefit studies of stem cell niches, including the ability to isolate the entire structure as a 3D explant amenable to in-depth characterization of progenitor cell-microenvironment interactions. Combining this with high-content imaging, we can examine critical components and structures across the whole NSC niche. Given that many of the features we want to analyze are multi-faceted, such as the V-SVZ vasculature plexus and the distribution of different progenitor cells with respect to the vasculature, we used software to digitize the composite montages, and then computer-based analysis of the thousands of datapoints per sample to provide unbiased data assessment. This approach enabled us to address some long-standing and important questions regarding whether the niche is sexually dimorphic and whether aging influences sex differences in NPC behavior and niche structure. We have discovered complex age-related changes in the niche that are sex dependent. Most notably, male mice showed a more dramatic, detrimental response to aging in the niche, with V-SVZ blood vessels becoming narrower, more tortuous, and dense, along with greater loss of NSCs and greater impairment of progenitor proliferation and migration than observed in females. This study demonstrates that aging affects both the vascular niche and resident NPCs differently in males and females, and underscores the importance of addressing the interaction of biological variables, such as sex and age in studies of stem cell regulation.

We discovered dramatic sex and age differences in the V-SVZ vasculature, which is known to regulate critical aspects of NSC maintenance, lineage progression, and migration of the cell products out of the niche. Sex hormones, which decline with aging, alter the function of blood vessels in the brain (Robison et al., 2019; Zuloaga et al., 2014), and both endothelial cells and vascular smooth muscle cells express sex hormone receptors (Zuloaga et al., 2012a, 2012b). Estrogens decrease brain-blood vessel tone and increase blood flow, angiogenesis, and blood-brain barrier function, and reduce inflammation and oxidative stress. Androgen actions are more complicated and often dose/duration/age dependent, but in general, chronic androgen administration has pro-angiogenic and vasoconstrictor effects. As sex hormone levels decrease with age, the vascular response is likely to be sexually dimorphic and this in turn is expected to impact the niche and NPC behavior.

Estrogen loss decreases cerebral cortical capillary density (Jesmin et al., 2003). Consistent with this, we found a decrease in vessel density in the V-SVZ of 22-month-old females. Data on androgen effects in cerebral vessels is lacking. Here, we show that V-SVZ vessel density increases by 22 months in males. Vascular density has previously been reported to decline with age in males (Katsimpardi et al., 2014); however, this was calculated from 100-μm-thick coronal brain sections, which informs about a more restricted region, while our studies examined vascular structure across the entire niche in 3D, providing a global view that shows an increase in vessel density.

Vessel tortuosity increases with age in a variety of vascular beds, including the cerebral vasculature, and is increased in many disease states (Han, 2012). Severe increases in tortuosity can even restrict blood flow to the point of ischemia (Wang et al., 2016). The V-SVZ vasculature is unique in that the vessels form a dense vascular plexus running largely parallel to the ependymal surface that is easily distinguishable from the surrounding tissue by its highly linear organization (Shen et al., 2008). Tortuosity of vessels is low in the V-SVZ and increases further from the lateral ventricle wall (Culver et al., 2013). We found significantly increased tortuosity with age in male but not female V-SVZ blood vessels. Since increased tortuosity is a departure from the normal vessel structure, this change could adversely affect blood flow, resulting in the reduced flow reported in the V-SVZ in males (Katsimpardi et al., 2014).

Blood vessel lumen diameter is inversely proportional to blood flow. Although the V-SVZ is reported to have lower blood flow than surrounding tissues, vessel diameter has not previously been reported. We found that V-SVZ vessel diameter decreased with age in males, but increased between 18 and 22 months in females, including the gain of a second population of larger capillaries (>8.25 μm). This resulted in aged males having much smaller vessel diameters than aged females. Whether vessel diameter changes resulted in changes in lumen size, which are predicted to affect blood flow, is not clear. It is possible that excessive thickening of the basement membrane, which occurs in other vessels in aged/acyclic female rodents (Gerrits et al., 2010), contributes to the increased diameter in aged females; an increase in basement membrane thickness could cause vessel rigidity. Another possibility that aligns with the less-severe age-related decrease in NPC changes, is that the female capillary lumens widened with age to compensate for a decrease in vessel density. It is unclear what the net effect of these vessel changes would have on blood flow in the aging V-SVZ; in the future, measures of blood flow in males versus females would be worthwhile. Overall, our data suggest that males and females may compensate for age-related vascular changes in different ways: males may compensate for an age-associated increase in narrow, tortuous vessels by increasing density, while females may compensate for a decrease in density by increasing vessel diameter.

V-SVZ blood vessels guide neuroblast migration, and therefore we examined neuroblast chain structure with age. The density of DCX+ chains declined with age, particularly in the anterior V-SVZ, in both ages. However, the chains of DCX+ neuroblasts became more disorganized with age in males but not females. In young animals, DCX+ neuroblasts are within approximately 6 μm of the nearest vessel surface. With advanced age, male neuroblasts get closer to the vessel surface, while in females neuroblasts move further away. How this situation arises is not clear but could be related to the dramatic changes in blood vessel structure. By 22 months, males have increased vessel density and increased vessel tortuosity, so normal neuroblast guidance and migration might be disrupted, leading to the DCX+ cells being trapped close to the vasculature. In contrast, in females by 22 months, the vessels have become less dense, of larger diameter, and less torturous, so there may be fewer vessels near existing chains and migration of DCX+ cells away from the vessels might be facilitated.

With aging, the NSC population declines, as does progenitor proliferation in the V-SVZ niche (Ahlenius et al., 2009; Conover and Shook, 2011; Jin et al., 2003; Luo et al., 2006); however, previous V-SVZ aging studies have been performed on males. We were surprised to find that the decline in NSC number and progenitor proliferation is significant in males but not in females. Even in the anterior domain, which has significant NSC and neurogenic activity, proliferation did not change with aging in females. Consistent with previous reports, we did not detect sex differences in 2-month-old mice (Tatar et al., 2013), although there was a trend toward increased proliferation in males, previously seen in 3.3-month-old mice (Diaz et al., 2009). We did not examine 6- to 8-month-old mice, when twice as much proliferation has been reported in the V-SVZ in females than males (Tatar et al., 2013). Given the differential effects we see across the sexes at 2, 18, and 22 months, understanding niche composition across a finer time course would be valuable. Overall, our studies indicate that NSCs and their lineage progeny are more vulnerable to aging in males than in females.

Several studies support a role for adult sex steroids on regulating neurogenesis. NPC proliferation increases in the V-SVZ of female rodents during phases of the estrus cycle when estradiol levels are highest, or when exogenous estradiol is administered (Mazzucco et al., 2006; Smith et al., 2001; Tanapat et al., 1999; Tatar et al., 2013). Castration in young males decreased proliferation in the V-SVZ, particularly of MASH-1+ type C cells, while replacement with estradiol or testosterone restored their proliferation (Farinetti et al., 2015). Most studies have been conducted in young rodents, although one study showed that estradiol promotes proliferation in the dentate gyrus, another adult NSC niche, in aged females (Perez-Martin et al., 2005). Furthermore, there may be benefit from sex hormone treatment to counteract the impact of stressors associated with aging: estrogen increases proliferation of both male and female V-SVZ progenitors after interferon gamma treatment *in vitro* and estrogen protects the cells from UV-induced cell death (Kim and Casaccia-Bonnefil, 2009). Hence, local activation of sex hormone receptors could help reduce the impact of aging on NPC behavior in the niche, but our study, along with prior work, indicates the type of intervention required would likely vary with both sex and age.

In conclusion, this study provides novel information regarding the complex, sex-dependent changes in the V-SVZ niche with age. Specifically, we demonstrate that the male V-SVZ vascular niche shows greater age-related vascular changes and proliferation deficits than the female V-SVZ at 22 months. These sex differences could emanate from developmental differences, activational effects of hormones in adulthood, or sex chromosome or X-inactivation effects. In addition to considering the impact of gonadal hormone reduction with age, we note that sex hormones can be produced locally in the vasculature and the brain (Hajszan et al., 2007; Hojo et al., 2004; McEwen et al., 2016), and brain endothelial cells express aromatase that can produce estradiol in both sexes (Zuloaga et al., 2014). Therefore, age-dependent changes in these local sources deserve further investigation, as do changes in the various receptors to progesterone, androgens, and estrogens that exhibit differential expression on brain cell subtypes (Contreras-Zárate and Cittelly, 2020). It will also be worthwhile exploring whether similar sexual dimorphism of the periventricular region or other zones that harbor NPCs in humans (Nicaise et al., 2020; Seki, 2020), occurs with aging, such as altered vascular structure and abnormal progenitor proliferation (Matarredona and Pastor, 2019), as these could contribute to different courses in age-related brain diseases in men and women.

## Experimental procedures

### Animals

Experiments were approved by the SUNY Albany IACUC. C57BL/6 mice were obtained from the National Institute of Aging aged mouse colony. V-SVZ whole mounts were dissected from male and female mice at age 2, 18, and 22 months (n = 4–6 per group). The whole-mount studies were performed in 9 separate cohorts, each of 3–6 mice. Time-lapse experiments were conducted on 4 cohorts of 12–18 mice (3 mouse brains were pooled for each replicate). To reduce cohort effects, each cohort included male and female and at least 2 ages, and each group was run with each other group during at least one of the cohorts. Mice were housed for at least 2 weeks before imaging.

### Whole-Mount Preparation

Mice were anesthetized with isoflurane and then perfused with saline for 5 min with pressure at 60–70 mm Hg. The V-SVZ was dissected as described (Shen et al., 2008), then fixed in ice-cold methanol for 10 min, and rinsed 3 times with PBS before staining. One V-SVZ from each mouse (right or left hemisphere chosen at random) was selected for immunolabeling. For immunohistochemistry, imaging, and analysis details see the Supplemental information.

### NPC Isolation and Live-Cell Time-Lapse Imaging and Analysis

NPCs were isolated from V-SVZs as described previously (Apostolopoulou et al., 2017), see supplemental information. V-SVZs from 3 mice were pooled, the cells dissociated and plated in 4 wells of a 24-well plate coated with poly-L-ornithine (Sigma, cat. no. 4957). Cells were cultured with DMEM-based medium (see supplemental experimental procedures). Each experiment was repeated 3–4 times (n = 9–12 mice per group/12–16-well per group).

A total of 315 movies were analyzed. The movies were captured in four different experiments on different dates. Each movie contained cells from a different sex and age (2, 18, and 22 months). See Supplemental information for segmentation, tracking, and population slope methods.

### Statistics

For vessel tortuosity, vessel diameter, and DCX chain eccentricity data, p values were calculated by the Wilcoxon ranked sum difference of the median. For vessel density, DCX area covered, Ki67 counts, *in vitro* GFAP cell quantification and population slopes, data were analyzed by two-way ANOVA (age × sex) with Sidak's post hoc multiple comparisons test. For vessel diameter distribution, p values were calculated by the Kolmogorov-Smirnov test. For whole-mount V-SVZ GFAP quantification, distance measurements between GFAP- or DCX-stained cells and blood vessels, data were analyzed by two-way ANOVA (age × sex) with Tukey's post hoc multiple comparisons test.

### Data Presentation

Data presented as the median ± 95% confidence intervals for vessel tortuosity, vessel diameter, and neuroblast chain eccentricity. Data presented as mean ± SEM for vessel density, DCX area, Ki67+ cell counts, GFAP-related cell quantification and population slopes.

### Data and Code Availability

The image data utilized in this project requires over 4TB of storage and is available on request. The source code as described in the image analysis section is a customized version of the open-source LEVER software tools. For more details see https://leverjs.net.

## Author contributions

S.T., K.L.Z., C.S.B., X.Z., and A.R.C. designed the experiments. C.S.B., K.L.Z., and Y.W. collected, immunolabeled, and imaged the whole mounts. K.L.Z. and Y.W. performed the time-lapse experiments. X.Z., E.W., W.M., K.L.Z., and A.R.C. analyzed the data. S.T., K.L.Z., A.R.C., Y.W., X.Z., and E.W. interpreted the data. K.L.Z., S.T., and X.Z. prepared the manuscript. S.T., K.L.Z., A.R.C., and X.Z. edited the manuscript. All authors approved the manuscript.

## Supplemental information

Document S1. Supplemental experimental procedures and Figures S1 and S2

Document S2. Article plus Supplemental information

## Footnotes

Supplemental information can be found online at https://doi.org/10.1016/j.stemcr.2021.03.012.

## References

[bib1] Ahlenius H., Visan V., Kokaia M., Lindvall O., Kokaia Z. (2009). Neural stem and progenitor cells retain their potential for proliferation and differentiation into functional neurons despite lower number in aged brain. J. Neurosci..

[bib2] Apostolopoulou M., Kiehl T.R., Winter M., Cardenas De La Hoz E., Boles N.C., Bjornsson C.S., Zuloaga K.L., Goderie S.K., Wang Y., Cohen A.R. (2017). Non-monotonic changes in progenitor cell behavior and gene expression during aging of the adult V-SVZ neural stem cell niche. Stem Cell Reports.

[bib3] Apple D.M., Solano-Fonseca R., Kokovay E. (2017). Neurogenesis in the aging brain. Biochem. Pharmacol..

[bib4] Barron A.M., Pike C.J. (2012). Sex hormones, aging, and Alzheimer's disease. Front. Biosci. (Elite Ed.).

[bib5] Bovetti S., Hsieh Y.C., Bovolin P., Perroteau I., Kazunori T., Puche A.C. (2007). Blood vessels form a scaffold for neuroblast migration in the adult olfactory bulb. J. Neurosci..

[bib6] Capilla-Gonzalez V., Cebrian-Silla A., Guerrero-Cazares H., Garcia-Verdugo J.M., Quinones-Hinojosa A. (2014). Age-related changes in astrocytic and ependymal cells of the subventricular zone. Glia.

[bib7] Codega P., Silva-Vargas V., Paul A., Maldonado-Soto A.R., Deleo A.M., Pastrana E., Doetsch F. (2014). Prospective identification and purification of quiescent adult neural stem cells from their in vivo niche. Neuron.

[bib8] Conover J.C., Shook B.A. (2011). Aging of the subventricular zone neural stem cell niche. Aging Dis..

[bib9] Contreras-Zárate M.J., Cittelly D.M. (2020). Sex steroid hormone function in the brain niche: implications for brain metastatic colonization and progression. Cancer Rep..

[bib10] Coskun V., Falls D.L., Lane R., Czirok A., Luskin M.B. (2007). Subventricular zone neuronal progenitors undergo multiple divisions and retract their processes prior to each cytokinesis. Eur. J. Neurosci..

[bib11] Culver J.C., Vadakkan T.J., Dickinson M.E. (2013). A specialized microvascular domain in the mouse neural stem cell niche. PLoS One.

[bib12] Diaz D., Valero J., Airado C., Baltanas F.C., Weruaga E., Alonso J.R. (2009). Sexual dimorphic stages affect both proliferation and serotonergic innervation in the adult rostral migratory stream. Exp. Neurol..

[bib13] Farinetti A., Tomasi S., Foglio B., Ferraris A., Ponti G., Gotti S., Peretto P., Panzica G.C. (2015). Testosterone and estradiol differentially affect cell proliferation in the subventricular zone of young adult gonadectomized male and female rats. Neuroscience.

[bib14] Gerrits P.O., de Weerd H., van der Want J.J., Kortekaas R., Luiten P.G., Veening J.G. (2010). Microvascular changes in estrogen-alpha sensitive brainstem structures of aging female hamsters. Neurosci. Res..

[bib15] Hajszan T., MacLusky N.J., Johansen J.A., Jordan C.L., Leranth C. (2007). Effects of androgens and estradiol on spine synapse formation in the prefrontal cortex of normal and testicular feminization mutant male rats. Endocrinology.

[bib16] Han H.C. (2012). Twisted blood vessels: symptoms, etiology and biomechanical mechanisms. J. Vasc. Res..

[bib17] Hojo Y., Hattori T.A., Enami T., Furukawa A., Suzuki K., Ishii H.T., Mukai H., Morrison J.H., Janssen W.G., Kominami S. (2004). Adult male rat hippocampus synthesizes estradiol from pregnenolone by cytochromes P45017alpha and P450 aromatase localized in neurons. Proc. Natl. Acad. Sci. U S A.

[bib18] Jesmin S., Hattori Y., Sakuma I., Liu M.Y., Mowa C.N., Kitabatake A. (2003). Estrogen deprivation and replacement modulate cerebral capillary density with vascular expression of angiogenic molecules in middle-aged female rats. J. Cereb. Blood Flow Metab..

[bib19] Jin K., Sun Y., Xie L., Batteur S., Mao X.O., Smelick C., Logvinova A., Greenberg D.A. (2003). Neurogenesis and aging: FGF-2 and HB-EGF restore neurogenesis in hippocampus and subventricular zone of aged mice. Aging Cell.

[bib20] Katsimpardi L., Litterman N.K., Schein P.A., Miller C.M., Loffredo F.S., Wojtkiewicz G.R., Chen J.W., Lee R.T., Wagers A.J., Rubin L.L. (2014). Vascular and neurogenic rejuvenation of the aging mouse brain by young systemic factors. Science.

[bib21] Kim J.Y., Casaccia-Bonnefil P. (2009). Interplay of hormones and p53 in modulating gender dimorphism of subventricular zone cell number. J. Neurosci. Res..

[bib22] Kokovay E., Goderie S., Wang Y., Lotz S., Lin G., Sun Y., Roysam B., Shen Q., Temple S. (2010). Adult SVZ lineage cells home to and leave the vascular niche via differential responses to SDF1/CXCR4 signaling. Cell Stem Cell.

[bib23] Lacar B., Herman P., Hartman N.W., Hyder F., Bordey A. (2012). S phase entry of neural progenitor cells correlates with increased blood flow in the young subventricular zone. PLoS One.

[bib24] Luo J., Daniels S.B., Lennington J.B., Notti R.Q., Conover J.C. (2006). The aging neurogenic subventricular zone. Aging Cell.

[bib25] Matarredona E.R., Pastor A.M. (2019). Neural stem cells of the subventricular zone as the origin of human glioblastoma stem cells. Therapeutic implications. Front. Oncol..

[bib26] Mazzucco C.A., Lieblich S.E., Bingham B.I., Williamson M.A., Viau V., Galea L.A. (2006). Both estrogen receptor alpha and estrogen receptor beta agonists enhance cell proliferation in the dentate gyrus of adult female rats. Neuroscience.

[bib27] McEwen B.S., Nasca C., Gray J.D. (2016). Stress effects on neuronal structure: hippocampus, amygdala, and prefrontal cortex. Neuropsychopharmacology.

[bib28] Mirzadeh Z., Merkle F.T., Soriano-Navarro M., Garcia-Verdugo J.M., Alvarez-Buylla A. (2008). Neural stem cells confer unique pinwheel architecture to the ventricular surface in neurogenic regions of the adult brain. Cell Stem Cell.

[bib29] Nicaise A.M., Willis C.M., Crocker S.J., Pluchino S. (2020). Stem cells of the aging brain. Front. Aging Neurosci..

[bib30] Ottone C., Krusche B., Whitby A., Clements M., Quadrato G., Pitulescu M.E., Adams R.H., Parrinello S. (2014). Direct cell-cell contact with the vascular niche maintains quiescent neural stem cells. Nat. Cell Biol.

[bib31] Perez-Martin M., Salazar V., Castillo C., Ariznavarreta C., Azcoitia I., Garcia-Segura L.M., Tresguerres J.A. (2005). Estradiol and soy extract increase the production of new cells in the dentate gyrus of old rats. Exp. Gerontol..

[bib32] Ponti G., Farinetti A., Marraudino M., Panzica G., Gotti S. (2018). Sex steroids and adult neurogenesis in the ventricular-subventricular zone. Front. Endocrinol. (Lausanne).

[bib33] Robison L.S., Gannon O.J., Salinero A.E., Zuloaga K.L. (2019). Contributions of sex to cerebrovascular function and pathology. Brain Res..

[bib34] Seki T. (2020). Understanding the real state of human adult hippocampal neurogenesis from studies of rodents and non-human primates. Front. Neurosci..

[bib35] Shen Q., Wang Y., Kokovay E., Lin G., Chuang S.M., Goderie S.K., Roysam B., Temple S. (2008). Adult SVZ stem cells lie in a vascular niche: a quantitative analysis of niche cell-cell interactions. Cell Stem Cell.

[bib36] Silva-Vargas V., Maldonado-Soto A.R., Mizrak D., Codega P., Doetsch F. (2016). Age-dependent niche signals from the choroid plexus regulate adult neural stem cells. Cell Stem Cell.

[bib37] Smith M.T., Pencea V., Wang Z., Luskin M.B., Insel T.R. (2001). Increased number of BrdU-labeled neurons in the rostral migratory stream of the estrous prairie vole. Horm. Behav..

[bib38] Solano Fonseca R., Mahesula S., Apple D.M., Raghunathan R., Dugan A., Cardona A., O'Connor J., Kokovay E. (2016). Neurogenic niche microglia undergo positional remodeling and progressive activation contributing to age-associated reductions in neurogenesis. Stem Cells Dev..

[bib39] Tanapat P., Hastings N.B., Reeves A.J., Gould E. (1999). Estrogen stimulates a transient increase in the number of new neurons in the dentate gyrus of the adult female rat. J. Neurosci..

[bib40] Tatar C., Bessert D., Tse H., Skoff R.P. (2013). Determinants of central nervous system adult neurogenesis are sex, hormones, mouse strain, age, and brain region. Glia.

[bib41] Tavazoie M., Van der Veken L., Silva-Vargas V., Louissaint M., Colonna L., Zaidi B., Garcia-Verdugo J.M., Doetsch F. (2008). A specialized vascular niche for adult neural stem cells. Cell Stem Cell.

[bib42] Thore C.R., Anstrom J.A., Moody D.M., Challa V.R., Marion M.C., Brown W.R. (2007). Morphometric analysis of arteriolar tortuosity in human cerebral white matter of preterm, young, and aged subjects. J. Neuropathol. Exp. Neurol..

[bib43] Wang L., Zhao F., Wang D., Hu S., Liu J., Zhou Z., Lu J., Qi P., Song S. (2016). Pressure drop in tortuosity/kinking of the internal carotid artery: simulation and clinical investigation. Biomed. Res. Int..

[bib44] Zuloaga K.L., Davis C.M., Zhang W., Alkayed N.J. (2014). Role of aromatase in sex-specific cerebrovascular endothelial function in mice. Am. J. Physiol. Heart Circ. Physiol..

[bib45] Zuloaga K.L., O'Connor D.T., Handa R.J., Gonzales R.J. (2012). Estrogen receptor beta dependent attenuation of cytokine-induced cyclooxygenase-2 by androgens in human brain vascular smooth muscle cells and rat mesenteric arteries. Steroids.

[bib46] Zuloaga K.L., Swift S.N., Gonzales R.J., Wu T.J., Handa R.J. (2012). The androgen metabolite, 5alpha-androstane-3beta,17beta-diol, decreases cytokine-induced cyclooxygenase-2, vascular cell adhesion molecule-1 expression, and P-glycoprotein expression in male human brain microvascular endothelial cells. Endocrinology.

