# Supplementary material for: 3D Image Analysis of the Complete Ventricular-Subventricular Zone Stem Cell Niche Reveals Significant Vasculature Changes and Progenitor Deficits in Males Versus Females with Aging
Source: Stem Cell Reports. 2021 Apr 8;16(4):836–50. doi: 10.1016/j.stemcr.2021.03.012 (PMC8072131; doi:10.1016/j.stemcr.2021.03.012)
Supplement: Document S1. Supplemental experimental procedures and Figures S1 and S2 [file mmc1.pdf]

Stem Cell Reports, Volume *16*

Supplemental Information

3D Image Analysis of the Complete Ventricular-Subventricular Zone Stem Cell Niche Reveals Significant Vasculature Changes and Progenitor Deficits in Males Versus Females with Aging

Xiuli Zhao, Yue Wang, Eric Wait, Walt Mankowski, Christopher S. Bjornsson, Andrew R. Cohen, Kristen L. Zuloaga, and Sally Temple

SUPPLEMENTAL INFORMATION

SUPPLEMENTAL TABLE OF CONTENTS

Supplemental Figures and Legends

- **Figure S1. V-SVZ Vascular Changes with Age.**
- **Figure S2. Schematic to illustrate the approach used to examine the distance of NPCs to the nearest blood vessel surface with quantification using Imaris distance transformation (Bitplane).**

Supplemental Experimental Procedures

Supplemental References

Supplemental Figures and Legends

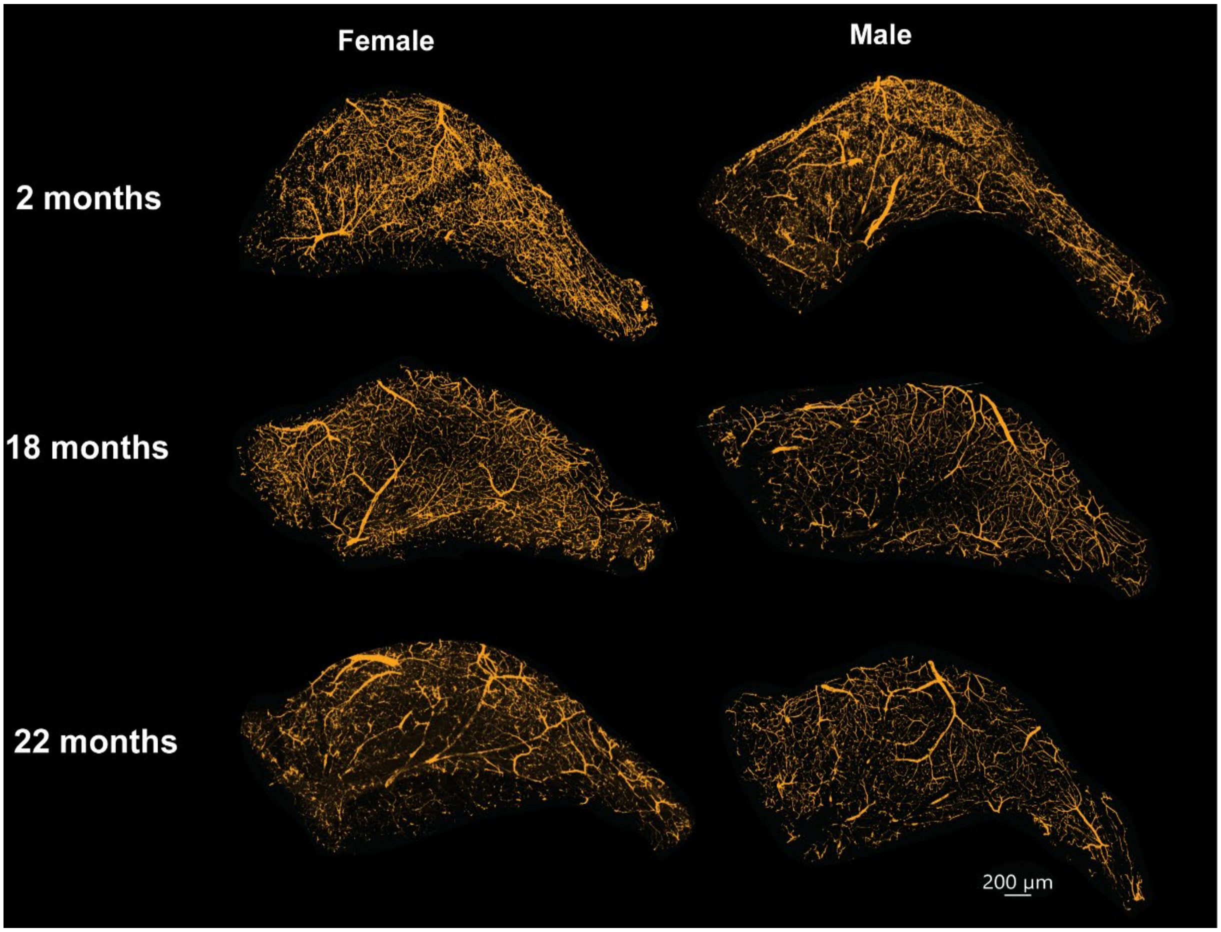

Figure S1. V-SVZ Vascular Changes with Age. Representative images are shown for V-SVZ wholemounts labeled with laminin to reveal blood vessels. Each 3D montage (approximately 50GB) was generated from about 50 individual 3D images that were stitched together. Scale bar 200uM.

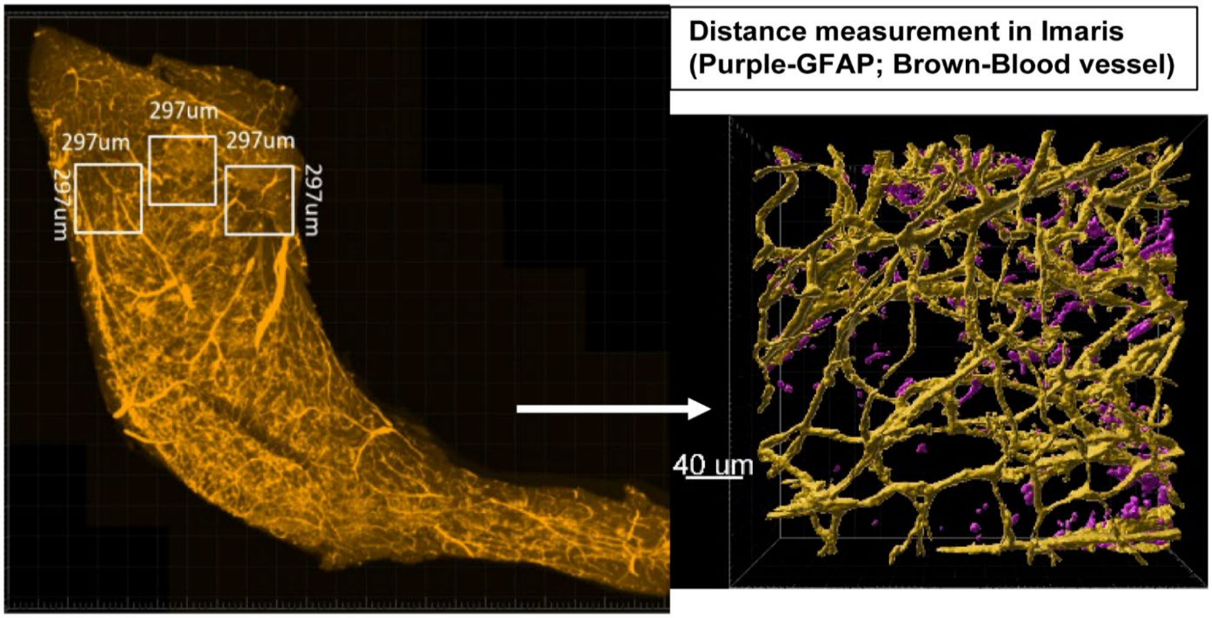

Figure S2. Schematic to illustrate the approach used to examine the distance of NPCs to the nearest blood vessel surface with quantification using Imaris distance transformation (Bitplane). We cropped 3 regions (X, Y=297,297uM) from the anterior V-SVZs of each mouse. We combined the data from the distance measurement of these regions for each mouse and performed statistics in Graphpad 8 (Prism).

Supplemental Experimental Procedures

Animals: Protocols were approved by the SUNY Albany IACUC. C57BL/6 mice were obtained from the NIA aged mouse colony. V-SVZ wholemounts were dissected from male and female mice at 2, 18, and 22 months of age (n=4-6 per group, see figure legends). The wholemount studies were performed with 9 cohorts of 3-6 mice. Time lapse experiments were conducted in 4 cohorts of 12-18 mice (3 mouse brains were pooled for each replicate). For both types of experiments, each cohort included two sexes and at least 2 ages. Each group was run alongside each other group during at least one of the cohorts. Mice were housed in the animal facility for at least two weeks before imaging. All females were virgins and group-housed throughout their lifespan (2-5 mice per cage). Estrus cycle was not determined; however, by 18 months of age females are acyclic (Mobbs et al., 1984). Mice were perfused with saline for 5 min with pressure maintained at 60-70mmHg prior to V-SVZ dissection. Exclusion criteria included: torn wholemount tissue (which can occur during dissection), inability to threshold the image due to poor labeling, weight loss > 10%, severe dermatitis, or tumors.

Wholemount preparation and Immunolabelling: The V-SVZ was dissected from the brains as previously described (Shen et al., 2008), then fixed in ice-cold methanol for 10 min, and rinsed 3 times with PBS prior

to staining. One V-SVZ from each mouse (right or left hemisphere chosen at random) was selected for immunolabeling with one of two panels. Panel 1: Wholemounts were incubated in Dako Protein Block (1hr), then 0.5% triton-X in phosphate buffered saline (TPBS) (1hr). Primary antibodies were incubated overnight at 4°C: goat anti-doublecortin (DCX; 1:300; Santa Cruz Cat# sc-8066), rat anti-laminin (1:500; Sigma Cat# L0663), and rabbit anti-Ki67 (1:200; Thermo Cat# PAS-19462). Following 3 washes with 0.5% TPBS, V-SVZ wholemounts were labeled with secondary antibodies: donkey anti-goat IgG 488 (1:300; Jackson Immuno), donkey anti-rat IgG CY3 (1:300; Jackson Immuno), donkey anti-rabbit IgG 514 (1:500; Jackson Immuno), and DAPI (1:1,000; Cat# D1306, Invitrogen). After incubation overnight at 4°C, the wholemounts were washed 3 times with 0.5% TPBS then mounted on glass slides with tissue spacers in Prolong Gold Antifade reagent with DAPI (Invitrogen, Cat# P366931). Panel 2: Wholemounts were incubated in 0.5%TPBS (1hr), then 10% normal donkey serum (NDS; 1h). Primary antibodies were incubated overnight at 4°C: mouse anti-beta catenin (1:200; BD Transduction Lab 610154) and chicken anti-GFAP (1:300; Aves) overnight @ 4 °C in 10%NDS. Following 3 washes with 0.5% TPBS, V-SVZ wholemounts were labeled with secondary antibodies (all from Jackson Immuno): anti-donkey anti-mouse IgG Cy3(1:300), donkey anti-chicken IgY 594(1;200) and DAPI (1:1000) overnight at 4°C in 10% NDS. Next, the wholemounts were washed 3 times with 0.5% TPBS then mounted on glass slides with tissue spacers in Prolong Gold Antifade reagent with DAPI (Invitrogen, Cat# P366931).

Imaging: Images were collected on a Zeiss LSM780 microscope in multitrack channel mode using a Plan-Apochromat 10x/0.45 objective. Widefield fluorescence was first employed to determine the brightest signal for each channel across all specimens stained during each immunofluorescent run. These brightest regions were then used to optimize confocal settings to ensure all samples fell within the dynamic range of the GaAsP PMT detectors. Z-stack images were collected using ZEN Black software used the following settings: frame size 2048 x 2048, averaging 8 frames, 3 micron Z-step, and were collected as tiled 3D montages with 10% overlap using the convex hull tiling approach. Single positives were collected using identical settings to generate fingerprints representing each fluorophore across the different channels using custom unmixing software.

V-SVZ image registration: V-SVZ image stacks were registered to form a single large montage image as described previously (Wait et al., 2014), with a normalized covariance score establishing best correspondences between imaging stacks, and stacks were combined using a maximum spanning tree approach.

DCX/Ki67 segmentation and quantification: Linear unmixing was performed (Zimmermann, 2005). Background was removed using a high-pass filter (Wait et al., 2014). For both the DCX+ and Ki67 channels, a Laplacian of Gaussian filter was used to identify edges and bright interiors. Connected component analysis (Gonzales, 2009) was used to identify objects, migrating neuroblast chains in the DCX channel

and number of proliferative cells in the Ki67 channel. To identify V-SVZ anterior regions in a manner unbiased by size and shape, the images were placed on a common 3D coordinate set using a medial axis transform (Sherbrooke, 1996) to create an X axis running anterior to posterior. The Y positions are calculated as the distance above (dorsal) or below (ventral) the medial axis transform. Anterior objects are then found with X coordinates greater than 0.25.

Vessel segmentation and quantification in V-SVZ montage images: Vessel skeletonization was done using a MATLAB toolbox for 3-D vessel topological skeletonization (https://www.mathworks.com/matlabcentral/fileexchange/43400-skeleton3d) (Kerschnitzki, 2013). The SVZ images were reduced in size using the *imresize3d* command so that the maximal dimension was 2000 voxels, and rescaled so that each voxel was a cube. The resize was required due to maximum supported image size in Microsoft's DirectX used by the 3-D image viewer (Wait et al., 2014). The size reduction was also effective at smoothing (cubic interpolation) and noise reducing the vascular channel. For each skeleton segment, returned as part of the skeletonization graph, tortuosity was computed as the ratio of vessel path length (number of voxels) to the Euclidean distance between endpoints. Vessel lengths were computed by adding per voxel lengths across the direction of travel for the skeleton result, and vessels of length less than 50 microns were not considered. Thus, vessels were identified by thresholding of laminin labeling and by having a minimum length of 50 microns. There were no diameter exclusion criteria. Vessels ranged 1.35 to 22.35 microns in diameter. Density of vessels was calculated as the vessel volume related to the total V-SVZ volume.

NPC distance from vessel surface: After immunostaining for panel 1, using Imaris software (Bitplane), we created digital surfaces of the blood vessels, DCX+ and GFAP+ positive cells, and DAPI stained nuclei based on the fluorescent intensity value using images. Using Imaris surface colocalization XTension, we determined colocalized GFAP and DAPI and use them as GFAP cell nuclei. The Imaris Distance Transformation XTension function was used to calculate the distance in 3D space between the DCX cells or GFAP cell nuclei and the nearest blood vessel surface.

Apical type B cell quantification: After immunostaining panel 2, using Imaris software, we created digital surfaces of the beta-Catenin+ ependymal cells and the GFAP+ cells. The soma of the GFAP+ cells were identified by GFAP+ and DAPI+ overlap using the Imaris Distance Transformation XTension function, enabling us to assess thousands of cells in each image in an unbiased manner. We first calculated the distance between segmented GFAP+ stained cells and DAPI+ cell nuclei. If a DAPI+ nucleus was within 0uM of a GFAP+ stained cell, we defined this as colocalization. Following, we quantified the number of GFAP+ soma within 5um of the beta-catenin+ ependymal surface layer. The same parameters were applied to both males and females in each age category.

Neural Progenitor Cell Isolation and Culture: NPCs were isolated from V-SVZs as previously described (Apostolopoulou et al., 2017). V-SVZs from 3 mice were pooled together and plated in 4 wells of a 24 well

plate. Each experiment was repeated 3-4 times (n= 9-12 mice per group/12-16 well per group). Growth medium was DMEM, supplemented with 1 mM Na-pyruvate, 2 mM L-glutamine, 0.16 µg/mL N-acetyl-L-cysteine, N2 supplement, B27 supplement, 0.01 µg/mL FGF 2, 0.01 µg/mL BDNF, 0.05 µg/mL NRG1, 0.1 mM QVD, and 0.01 mM Y27632. Cells settled for ~2 hr before medium was changed to remove debris, just prior to timelapse imaging. Each 24-well plate of cells was imaged on a Zeiss Observer Z1 microscope equipped with a Colibri camera and an environmental chamber to maintain temperature at 37°C and CO_2 at 5% (Pecon XLS1 incubation). Five fields of vision with cells were chosen randomly from four wells per age group/experiment, totaling 20 fields/age group/experiment. Fields were imaged every 5 minutes. Imaging was paused once to allow for re-feeding of cells at 2 days in vitro (DIV) and experiments were ended at 4 DIV.

Live cell time-lapse image analysis: A total of 315 movies were analyzed. The movies were captured from four different experiments on different dates. Each experiment, and each movie set) included cells from a different age (2, 18, 22 months) and sex. Each image frame contains 1344x1024 pixels, and each movie consists of 980 image frames captured every five minutes. Image analysis is described in supplemental methods.

The movies were segmented and tracked using the LEVER program as described previously (Winter et al., 2016; Winter et al., 2011; Winter et al., 2015). We applied a new analytic approach to the segmentation and tracking results. Previous work has analyzed developmental dynamics of proliferating cells to cell cycle times or cell motion and morphology to identify statistically significant differences among cells and clones from different experimental populations (Apostolopoulou et al., 2017; Stadler et al., 2018; Winter et al., 2015), or have used semi-supervised machine learning techniques based on Kolmogorov complexity theory to identify meaningful differences in dynamic behaviors (Cohen, 2014; Cohen, 2009; Cohen et al., 2010; Cohen and Vitanyi, 2015). These approaches all rely on accurate tracking information; while it is generally possible to be robust to segmentation errors, even a single tracking error can corrupt subsequent analyses (Cohen, 2009).

Rather than relying on perfectly correct tracking, the approach applied here is to compute a robust "population slope" feature per movie. A linear regression fits the number of segmentations per image frame, generating a slope value representing population growth. This feature captures a course view of cell mitosis and death. The population slope feature will be impacted by cells exiting and leaving the frame, but such events are considered uniformly distributed across the experimental conditions. The population slope feature is then compared using statistical tests (two sample t-test) across ages and genders to identify statistically significant changes in proliferation.

Immunofluorescence labeling of V-SVZ cells after timelapse live imaging:

Cells were fixed in 4% paraformaldehyde/0.1MPHEM, at room temperature (RT) for 20 minutes, washed 3x with PBS, incubated with rabbit anti-EGFR primary antibody (1:200 diluted in 1XPBS, Thermofisher, Cat# PA1-1110) at 4 °C, overnight, then washed 3x with PBS before incubating with secondary goat anti-rabbit IgG546 (1:1000 diluted in 1XPBS, Invitrogen, Cat# A-11010) at RT for 30 minutes. After washing 3x in PBS, DAPI was added (1:1000 in PBS, Invitrogen, Cat# D-1306) and incubated at RT for 5 minutes. After 3x washes with PBS, cells were incubated at RT for 1hr with chicken IgY anti-GFAP primary antibody (1:400 diluted in PBS with 0.1% triton-X (PBS-TX), Aves Labs, Cat# GFAP), washed 3x with PBS and incubated with secondary goat anti-chicken 488 (1:1000 diluted in PBS-TX; Jackson Labs, Cat# 115-485-205) at RT for 30 minutes. Cells were washed 3x with PBS and incubated with mouse IgG2b anti-β-tubulin III antibody (1:500 diluted in PBS-TX, Sigma, Cat# T8660) for 1hr at RT, washed 3x with PBS then incubated with goat anti-mouse IgG2b 647(1:500 diluted in PBS-TX, Invitrogen, Cat#A-21242) at RT for 30 minutes. Finally, after washing 3x with PBS, the cells were imaged and the immunostaining was quantified.

β-tubulin III primary antibody (Sigma, Cat# T8660) was used for these analyses given its reliable staining of neurons in vitro, while DCX (Santa Cruz Cat# sc-8066) is preferred for tissue sections. Furthermore, given that DCX labels young migrating neuroblasts but is transient, while ß-tubulin III stains young neurons and is maintained, and because we wanted to capture all type A progeny in these in vitro studies, ß-tubulin III was the more reliable marker to use.

Supplemental References

Apostolopoulou, M., Kiehl, T.R., Winter, M., Cardenas De La Hoz, E., Boles, N.C., Bjornsson, C.S., Zuloaga, K.L., Goderie, S.K., Wang, Y., Cohen, A.R., *et al.* (2017). Non-monotonic Changes in Progenitor Cell Behavior and Gene Expression during Aging of the Adult V-SVZ Neural Stem Cell Niche. Stem Cell Reports *9*, 1931-1947.

Cohen, A.R. (2014). Extracting meaning from biological imaging data. Mol Biol Cell *25*, 3470-3473.

Cohen, A.R., Bjornsson, C., Temple, S., Banker, G., and Roysam, B. (2009). Automatic Summarization of Changes in Biological Image Sequences using Algorithmic Information Theory IEEE Trans Pattern Anal Mach Intell *31*, 1386-1403.

Cohen, A.R., Gomes, F.L., Roysam, B., and Cayouette, M. (2010). Computational prediction of neural progenitor cell fates. Nat Methods *7*, 213-218.

Cohen, A.R., and Vitanyi, P.M. (2015). Normalized Compression Distance of Multisets with Applications. IEEE Trans Pattern Anal Mach Intell *37*, 1602-1614.

Gonzales, R., Woods, R., and Eddins, S. (2009). Digital Image Processing Using MATLAB (Knoxville TN USA: Gatesmark Publishing).

Kerschnitzki, M., Kollmannsberger, P., Burghammer, M., Duda, G.N., Weinkamer, R., Wagermaier, W., and Fratzl, P (2013). Architecture of the osteocyte network correlates with bone material quality. J Bone Miner Res *28*, 1837-1845.

Mobbs, C.V., Gee, D.M., and Finch, C.E. (1984). Reproductive senescence in female C57BL/6J mice: ovarian impairments and neuroendocrine impairments that are partially reversible and delayable by ovariectomy. Endocrinology *115*, 1653-1662.

Shen, Q., Wang, Y., Kokovay, E., Lin, G., Chuang, S.M., Goderie, S.K., Roysam, B., and Temple, S. (2008). Adult SVZ stem cells lie in a vascular niche: a quantitative analysis of niche cell-cell interactions. Cell Stem Cell *3*, 289-300.

Sherbrooke, E.C., Patrikalakis, N.M., and Brisson, E (1996). An Algorithm for the Medial Axis Transform of 3D Polyhedral Solids. IEEE Transactions on Visualization and Computer Graphics *2*, 44-61.

Stadler, T., Skylaki, S., K, D.K., and Schroeder, T. (2018). On the statistical analysis of single cell lineage trees. J Theor Biol *439*, 160-165.

Wait, E., Winter, M., Bjornsson, C., Kokovay, E., Wang, Y., Goderie, S., Temple, S., and Cohen, A.R. (2014). Visualization and correction of automated segmentation, tracking and lineaging from 5-D stem cell image sequences. BMC Bioinformatics *15*, 328.

Winter, M., Mankowski, W., Wait, E., Temple, S., and Cohen, A.R. (2016). LEVER: software tools for segmentation, tracking and lineaging of proliferating cells. Bioinformatics *32*, 3530-3531.

Winter, M., Wait, E., Roysam, B., Goderie, S.K., Ali, R.A., Kokovay, E., Temple, S., and Cohen, A.R. (2011). Vertebrate neural stem cell segmentation, tracking and lineaging with validation and editing. Nat Protoc *6*, 1942-1952.

Winter, M.R., Liu, M., Monteleone, D., Melunis, J., Hershberg, U., Goderie, S.K., Temple, S., and Cohen, A.R. (2015). Computational Image Analysis Reveals Intrinsic Multigenerational Differences between Anterior and Posterior Cerebral Cortex Neural Progenitor Cells. Stem Cell Reports *5*, 609-620.

Zimmermann, T. (2005). Spectral imaging and linear unmixing in light microscopy. Adv Biochem Eng Biotechnol *95*, 245-265.
